# Acute Capillary Bronchitis

**Published:** 1901-03

**Authors:** 


### Acute Capillary Bronchitis.

The following mixture has been recommended by Whitla, in the "Manual of Therapeutics":

℞ Vini antimonialis....... ...............

Spiritua chloroformi........ ... .....aa ʒiv

Spiritus ammoniæ arom .............. ℥i

Liquoris ammonii acetatis............ ʒii

Aquæ, q. s. ad......................... ℥viii

M. Sig.: A tablespoonful every two hours.—*Journal American Medical Association.*

